# Sex and Age Differences in the Effects of Food Frequency on Metabolic Parameters in Japanese Adults

**DOI:** 10.3390/nu16172931

**Published:** 2024-09-02

**Authors:** Katsumi Iizuka, Kotone Yanagi, Kanako Deguchi, Chihiro Ushiroda, Risako Yamamoto-Wada, Kazuko Kobae, Yoshiko Yamada, Hiroyuki Naruse

**Affiliations:** 1 Department of Clinical Nutrition, Fujita Health University, Toyoake 470-1192, Japan; kanasakuran@gmail.com (K.D.); chihiro.ushiroda@fujita-hu.ac.jp (C.U.); risako.wada@fujita-hu.ac.jp (R.Y.-W.); 2 Food and Nutrition Service Department, Fujita Health University Hospital, Toyoake 470-1192, Japan; 3 Health Management Center, Fujita Health University, Toyoake 470-1192, Japan; yanagi-k@fujita-hu.ac.jp (K.Y.); kobae@fujita-hu.ac.jp (K.K.); yoshiko.yamada@fujita-hu.ac.jp (Y.Y.); hnaruse@fujita-hu.ac.jp (H.N.); 4 Department of Medical Laboratory Science, Fujita Health University Graduate School of Health Sciences, Toyoake 4470-1192, Japan

**Keywords:** dietary preference, food frequency, BMI, uric acid, HbA1c, eGFR, triglyceride, HDL-C, non-HDL-C

## Abstract

Owing to differences in dietary preferences between men and women, the associations between dietary intake frequency and metabolic parameters may differ between the sexes. A retrospective observational study of the checkup findings of 3147 Japanese individuals (968 men, 2179 women) aged 20–59 years was conducted to examine differences in dietary habits and associations between food frequency and blood parameters (eGFR, HbA1c, uric acid, and lipids) by sex and age. Males were more likely to consume meat, fish, soft drinks, and alcohol, whereas women were more likely to consume soybeans, dairy products, vegetables, fruits, and snacks. Multivariate linear regression models adjusted for age and BMI revealed that meat intake frequency was positively associated with HbA1c (β = 0.007, *p* = 0.03) and negatively associated with eGFR (β = −0.3, *p* = 0.01) only in males, whereas fish intake frequency was positively associated with eGFR (β = 0.4, *p* = 0.005) only in females. Egg and soy intake frequencies were positively and negatively associated with non-HDL-C (egg: β = 0.6, *p* = 0.02; soy: β = −0.3, *p* = 0.03) only in females. Alcohol consumption frequency was associated with uric acid (M: β = 0.06, *p* < 0.001; F: β = 0.06, *p* < 0.001) and HDL-C (M: β = 1.0, *p* < 0.001; F: β = 1.3, *p* < 0.001) in both sexes. Future research is needed to determine whether varying the emphasis of dietary guidance by sex and age group is effective, since the effects of dietary preferences on metabolic parameters vary by age and sex.

## 1. Introduction

Eating habits and food preferences affect cognitive function, learning, body size, and the development of lifestyle-related diseases [1,2,3,4,5,6,7,8,9,10,11,12]. Eating habits included the frequency of meals, meal content, time taken to eat a meal, and the number of times the food was chewed. Dietary variety has been reported to be associated with cognitive function, chronic kidney disease, dental health, and all-cause mortality [13,14]. Dietary variety also reflects diet quality. Therefore, the greater the dietary variety is, the more types of nutrients are consumed.

Food preferences are influenced by many factors, including culture, age, and sex. The relationship between food preference and sex has also been documented [3,4,5,6,7,8,9,10,11]. Men prefer red and processed meats, whereas women tend to prefer vegetables, whole grains, tofu, and dark chocolate with a high cacao content, which is consistent with healthier food choices [1]. Women prefer whole grains, vegetables, and salty foods, whereas men consume more meat [2]. In terms of sugar intake, women tend to consume more sugar in their diets than men do [15]. In contrast, men consumed more alcoholic beverages than women did. Age is also an important factor that affects food preferences. Older adults tend to consume fewer energy-dense sweets and fast foods and consume more energy-dilute grains, vegetables, and fruits. The daily volume of food and beverages also decreases with age [16]. Therefore, dietary counseling for patients should consider differences in dietary preferences based on sex and age.

Dietary education in the workplace has not yet been adequately implemented in Japan. In France, work engagement has a positive relationship with the daily consumption of healthy food items [17]. However, in recent years, the concept of human capital has spread throughout Japan, creating a climate where companies are actively involved in disease prevention. The concept of health management emphasizes the importance of sleep debt [18,19]. Sleep debt worsens glucose tolerance, decreases thyrotropin levels, elevates cortisol levels in the evening, and decreases sympathetic tone [20]. 

The Food Frequency Questionnaire on Food Groups (FFQg), which was specifically created for the Japanese population, has often been used in Japan [21]. However, it has been used without considering sex differences in food preferences. Sex- and age-specific advice on the consumption of these 10 foods would compensate for the shortcomings of the guidelines, which arise from sex and age differences in food preferences.

The aim of this study was to determine the effects of food frequency on metabolic parameters, in terms of sex and age. For three thousand individuals aged 20–59 years, we first compared the differences in dietary preferences by age and sex. To clarify whether sex differences in food preference affect glucose and lipid metabolism, we examined the associations between food preference and blood parameters. This study not only reveals differences in food preferences by sex and age among health checkup recipients but also provides dietary guidance on the basis of sex differences in eating habits.

## 2. Materials and Methods

### 2.1. Study Design and Participants

This retrospective cross-sectional observational study aimed to clarify the associations between food frequency and metabolic parameters with reference to sex and age. The target population consisted of 968 men and 2179 women who had undergone a medical checkup by 2023 and had responded to the dietary questionnaire. The physical data and food frequency questionnaire were provided by the healthcare center at our university in a fully anonymized form, such that they were depersonalized (accessed on 26 March 2024).

### 2.2. Food Frequency Questionnaire on Food Groups

During the medical examination, the FFQg was used for dietary questions [21]. The FFQg is one of the most widely used food intake frequency questionnaires in Japan [22,23,24,25]. The FFQg included information on the frequency of eating 10 different food types (meat, fish, eggs, dairy products, soya, green vegetables, soya, green vegetables, seaweed, fruits, potatoes, oils, and fats), the frequency of drinking sugar-sweetened coffee and tea, and the frequency of consuming soft sweets, colas, and other soft drinks and alcohol [21]. For the ten food types, the subjects simply listed how many times per week they had consumed each type of food. The number of times snacks, sugar-sweetened coffee/tea, soft drinks, and alcoholic beverages were consumed every 7 days. The snacks included rice crackers, potato chips, cookies, cakes, and candies. Soft drinks included sweetened sugar beverages such as cola, cider, and fruit juice.

### 2.3. Physical Examination

Age (years), sex, body mass index (BMI, kg/m^2^), waist circumference (cm), handgrip strength (kg), sleep duration (h), and blood-sampling data (glycated hemoglobin A1c (HbA1c,%), estimated glomerular filtration rate (eGFR, mL/min/cm^2^), uric acid (UA, mg/dL), triglycerides (TG, mg/dL), high-density lipoprotein cholesterol (HDL-C, mg/dL), total cholesterol (TC, mg/dL), and non-HDL-C, mg/dL)) at the health checkup were obtained from the Health Management Department in the form of anonymized data linked to the FFQg. Height, weight, waist circumference (cm), and handgrip strength (kg) were measured in the presence of a nurse. BMI was automatically calculated from height and weight. Sleep duration was self-monitored, and the subjects simply listed the average sleep duration per week (e.g., six hours). The plasma creatinine, UA, and lipid concentrations (TC, TG, and HDL-C) were measured via a Hitachi LABOSPECT008 (Hitachi High-Tech Corporation, Tokyo, Japan), and HbA1c was measured via an A1c HA-8190 (Arkray, Kyoto, Japan). The eGFR was automatically calculated via the plasma creatinine level, age, and sex. Non-HDL-C concentrations were calculated from the TC and HDL-C concentrations. Blood samples were collected from nonfasting participants at approximately 16:00–17:00. As this was a hospital staff medical checkup, it was set at 3:30 p.m. when the medical staff had finished seeing the outpatients. The data are presented as the means (SDs).

### 2.4. Statistical Analysis

For comparisons by sex, t tests or Mann–Whitney U tests were used to compare men and women. Similarly, each individual was grouped as 20–29 years old (yo), 30–39 yo, 40–49 yo, or 50–59 yo, and we compared the food frequency of 20–29 yo with those of other age groups. Comparisons of the frequency of intake of the ten foods among the age groups were performed via the Kruskal–Wallis test followed by the Bonferroni method. To determine the effects of the frequency of eating the 10 food types, the frequency of sugar and alcohol intake, and sleep duration on body size and blood-sampling data (HbA1c, eGFR, UA, TG, HDL-C, and non-HDL-C levels), multivariate linear regression models adjusted for age and BMI were constructed. The data are presented as the means ± standard deviations (SDs). Statistical significance was set at *p* < 0.05. Statistical analyses were performed via SPSS version 28.0.0.0 for Mac software (IBM Corp., Armonk, NY, USA).

## 3. Results

### 3.1. Background of the Study Sample

Among the 3345 individuals (1057 men and 2268 women) who underwent all of the health examinations, those who were 20–59 years old and fully answered the dietary questionnaire were included in the study. The total number of individuals evaluated in this study was 3147 (M: 968, F: 2179), and the average age of the individuals (mean ± SD) was 35.1 ± 11.3 years (M: 37.9 ± 10.9, F: 33.9 ± 11.2) (Table 1). In men, age (yo) (mean ± SD), BMI (mean ± SD), waist circumference (cm) (mean ± SD), and handgrip strength (kg) (mean ± SD) were significantly greater than those in females (23.3 ± 3.4 vs. 21.3 ± 3.3, *p* < 0.001; 81.2 ± 9.1 vs. 70.8 ± 9.1, *p* < 0.001; 41.1 ± 9.1 vs. 24.5 ± 7.1, *p* < 0.001, respectively). Blood HbA1c (%) (mean ± SD), TG (mg/dL) (mean ± SD), non-HDL-C (mg/dL) (mean ± SD), and UA (mg/dL) (mean ± SD) levels were greater in men than in women (5.46 ± 0.42 vs. 5.40 ± 0.35, *p* < 0.001; 131.5 ± 95.6 vs. 87.8 ±55.8, *p* < 0.001; 139.19 ± 33.83 vs. 124.33 ±29.91, *p* < 0.001; 6.0 ± 1.2 vs. 4.2 ± 0.9, *p* < 0.001, respectively). In contrast, HDL-C (mean ± SD) and the eGFR (mean ± SD) were significantly lower in men than in women (53.7 ± 12.4 vs. 65.8 ± 13.6, *p* < 0.001; 84.7 ± 15.8 vs. 92.8 ± 17.7, *p* < 0.001, respectively). In males, sleep duration (h) (mean ± SD) was significantly shorter (6.3 ± 1.0 vs. 6.4 ± 1.0, *p* = 0.001). The frequencies of eating meat and fish (time per week; mean ± SD) were significantly greater in males (8.8 ± 4.5 vs. 7.8 ± 4.1, *p* < 0.001; 3.8 ± 3.0 vs. 3.3 ± 2.6, *p* = 0.001, respectively). In contrast, the frequencies of eating soybeans, dairy products, vegetables, and fruits (times per week; mean ± SD) in males were significantly lower than those in females (5.5 ± 4.3 vs. 5.9 ± 4.3, *p* = 0.002; 2.9 ± 2.8 vs. 3.3 ± 2.8, *p* < 0.001; 8.9 ± 4.8 vs. 9.3 ± 4.7, *p* = 0.029; 2.7 ± 2.7 vs. 3.1 ± 2.5 *p* < 0.001, respectively). Despite significant differences, both men and women consumed meat and vegetables almost daily; fish, dairy products, and eggs once every two days (four times per week) and soybeans, seaweed, and potatoes once every three or four days (three times per week) (Table 1).

Finally, men consumed soft drinks and alcohol more frequently per week (1.9 ± 3.0 vs. 1.0 ± 2.3, *p* < 0.001; 1.7 ± 2.4 vs. 1.1 ± 1.9, *p* < 0.001, respectively). In contrast, the frequency of eating snacks was greater among women (9.4 ± 11.9 vs. 11.7 ± 12.0, *p* < 0.001) (Table 1). The frequency of drinking coffee/tea with sugar was not significantly different (0.9 ± 2.8 vs. 0.9 ± 2.7, *p* = 0.057). Sex differences in the consumption of beverages and snacks are related to preferences.

### 3.2. Effects of Sex and Age on Food Frequency

We then compared food frequency across age groups (20–29, 30–39, 40–49, and 50–59 years) divided by sex (Table 2). No differences in the frequency of meat or egg intake were found between the 30-, 39-, 40–49-, and 50–59-year-old age groups and the 20–29-year-old group in either men or women. According to the results of the Bonferroni correction after the Kruskal–Wallis test, the consumption of fish, soybeans, dairy products, seaweed, fruits, potatoes, and oils and fats significantly increased in the 40 to 49-year-old and 50 to 59-year-old groups for men and women compared with the 20 to 29-year-old group (men:fish: 3.3 (3.0) vs. 3.7 (2.9) vs. 4.7 (3.3), *p* < 0.01; soybeans: 4.8 (4.2) vs. 6.0 (4.4) vs. 5.9 (4.1), *p* < 0.01; dairy products: 2.5 (2.4) vs. 3.2 (2.9) vs. 3.4 (2.8), *p* < 0.01; seaweed: 1.4 (1.5) vs. 2.2 (2.5) vs. 2.6 (2.0), *p* < 0.001; fruits: 1.9 (2.2) vs. 2.7 (2.4) vs. 3.9 (2.9), *p* < 0.001 The consumption of vegetables significantly increased in the 50 to 59-year-old groups for men and in the 40 to 49-year-old and 50 to 59-year-old groups for women compared with the 20 to 29-year-old group (men: 8.0 (4.4) vs. 10.1 (5.2), *p* < 0.001; women: 8.8 (0.1) vs. 9.4 (0.2) vs. 10.7 (0.3), *p* < 0.001). In contrast, the consumption of meat and eggs did not differ between the 40 to 49-year-old and 50 to 59-year-old groups for women compared with the 20 to 29-year-old group (men: meat: 8.7 (4.0) vs. 9.1 (4.8) vs. 8.9 (4.8), *p* = 0.76; egg: 4.3 (2.8) vs. 4.3 (3.7) vs. 4.2 (2.9), *p* = 0.4; women: meat: 7.7 (0.1) vs. 8.3 (0.2) vs. 7.5 (0.2), *p* = 0.02; egg: 4.1 (0.1) vs. 4.3 (0.1) vs. 4.2 (0.1), *p* = 0.9). Thus, most food frequencies differ according to age and sex, with the exceptions of eggs and meat.

### 3.3. Effect of Age on the Frequency of Consuming Snacks, Soft Drinks, and Alcoholic Beverages

We examined the effects of age on the frequency of consumption of snacks, sugar-sweetened coffee/tea, soft drinks, and alcohol according to sex (Table 3). The frequency of eating snacks increased significantly among men and women 40–49 years and 50–59 years of age (men: 8.0 (10.0) vs. 10.9 (10.5) vs. 10.2 (9.0), *p* < 0.001; women: 10.5 (10.3) vs. 13.5 (14.7) vs. 14.8 (14.6), *p* < 0.001) (Table 3). In contrast, the habits of drinking sugar-sweetened coffee, tea, or soft drinks were not significantly affected by age. The frequency of alcohol consumption increased significantly in both men and women aged 40--49 and 50--59 years (men: 1.3 (2.4) vs. 2.3 (2.6) vs. 2.2 (2.5), *p* < 0.001; women: 0.8 (1.2) vs. 1.6 (2.3) vs. 1.9 (2.6), *p* < 0.001). Thus, the frequency of eating snacks and drinking alcohol significantly increased at 40–49 and 50–59 years of age (Table 3).

### 3.4. Effects of Food Intake and Sugar-Sweetened Drink, Snack, and Alcohol Consumption on BMI, Waist Circumference, and Grip Strength

With age and BMI as covariates, the frequency of food intake and the frequency of food intake as independent variables, and waist circumference and grip strength as dependent variables, we conducted a multivariate linear regression analysis to determine the effect of the frequency of preferred food intake on waist circumference and grip strength. In males, waist circumference was positively associated with age (0.09 (0.05, 0.12), *p* < 0.001), BMI (2.1 (2.0, 2.2), *p* < 0.001), and the frequency of oil and fat intake (*p* = 0.014) and negatively correlated with the frequency of soybean intake (−0.05 (−0.09, 0.02), *p* = 0.004). In females, waist circumference was positively associated with age (0.24 (0.21, 0.26), *p* < 0.001), BMI (1.66 (1.58, 1.75), *p* < 0.001), hours of sleep (0.29 (0.03, 0.54), *p* = 0.03), frequency of meat intake (0.09 (0.02, 0.16), *p* = 0.01), and frequency of egg intake (−0.17 (0.07, 0.30), *p* = 0.001) and negatively associated with the frequency of soybean intake (−0.34 (−0.17, 0.03), *p* = 0.007) (Table 4).

In males, handgrip strength was associated with age (−0.1 (−0.15, −0.04), *p* < 0.001) and BMI (0.7 (0.5,0.8), *p* < 0.001). In females, handgrip strength was associated with BMI (0.14 (0.05, 0.23), *p* = 0.003), the frequency of seaweed intake (0.23 (0.07, 0.4), *p* = 0.005), and alcohol intake (0.23 (0.06, 0.39), *p* = 0.007). Thus, the effects of the frequency of food intake on body size and grip strength were more potent in women than in men (Table 4).

### 3.5. Effects of Food Intake Frequency and Sugar-Sweetened Drink, Sweet Drink, and Alcohol Consumption on HbA1c, eGFR and UA

Next, we conducted multivariate analyses to clarify the effects of the frequency of food intake on HbA1c, eGFR, and UA levels. The HbA1c level in males was positively associated with age (β (95% CI): 0.011 (0.009, 0.014), *p* < 0.001), BMI (0.031 (0.024,0.038), *p* < 0.001), and the frequency of meat intake (0.007 (0.001,0.013), *p* = 0.03) and negatively associated with hours of sleep (−0.04 (−0.064, −0.016), *p* = 0.001). In females, HbA1c was positively associated with age (0.01 (0.009, 0.012), *p* < 0.001) and BMI (0.02 (0.016, 0.024), *p* < 0.001) and negatively associated with hours of sleep (−0.03 (−0.04, −0.01), *p* < 0.001) and the frequency of alcohol intake (−0.018 (−0.025, −0.01), *p* < 0.001). Thus, age, BMI, and sleep duration affected HbA1c levels in both men and women, whereas the frequency of meat intake and alcohol intake affected HbA1c levels only in men and women (Table 5).

eGFR in males was positively associated with seaweed intake (0.8 (0.3, 1.2), *p* = 0.004) and negatively correlated with age (−0.8 (−0.9, −0.7), *p* < 0.001), frequency of meat intake (−0.3 (−0.5, −0.1), *p* = 0.008), and frequency of potato intake (−0.9 (−1.4, −0.3), *p* = 0.004). In females, the eGFR was positively associated with the frequency of fish intake (0.4 (0.1, 0.7), *p* = 0.001) and negatively correlated with age (−0.82 (−0.89, −0.76); *p* < 0.01), frequency of dairy product intake (−0.3 (−4.80, −0.47), *p* = 0.02), and frequency of snack intake (−0.66 (−1.01, −0.31), *p* < 0.001) (Table 5).

UA in males was positively associated with BMI (0.11 (0.09, 0.13); *p* < 0.001) and the frequency of alcohol intake (0.06 (0.03, 0.09) *p* < 0.001). In females, UA was positively associated with BMI (0.084 (0.073, 0.096), *p* < 0.001), the frequency of soybean intake (0.013 (0.003, 0.023), *p* = 0.009), and the frequency of alcohol intake (0.06 (0.04, 0.08), *p* < 0.001) and negatively associated with the frequency of dairy product intake (−0.01 (−0.03, 0), *p* = 0.048) and the frequency of oil and fat intake (−0.01 (−0.02, −0.002), *p* = 0.01). Thus, BMI and alcohol consumption were positively correlated with UA levels. The frequency of soybean, dairy products, and oil and fat intake was positively associated with UA only in women, whereas the frequency of dairy products and oil and fat intake was negatively associated with UA (Table 5).

### 3.6. Effects of Food Intake Frequency and Sugar-Sweetened Drink, Sweet Drink, and Alcohol Consumption on Serum Lipid Levels

Finally, we performed a multivariate analysis to determine whether food intake frequency and sugar-sweetened drinks, snacks, or alcohol consumption influenced lipid metabolism. TG levels in males were positively associated with age (1.1 (0.5, 1.7), *p* < 0.001), BMI (7.6 (5.9, 9.4), *p* < 0.001), frequency of potato intake (5 (0.9, 9), *p* = 0.02), and frequency of alcohol intake (3 (0.5, 5.4), *p* = 0.02) (Table 6). In females, TG levels were positively associated with age (1.2 (1.0, 1.4), *p* < 0.001), BMI (4.4 (3.7, 5.0), *p* < 0.001), frequency of meat intake (0.6 (0.04, 1.2), *p* = 0.04), and frequency of potato intake (1.8 (0.3, 3.2), *p* = 0.02) and negatively associated with the frequency of oil and fat intake (−0.7 (−1.2, −0.3), *p* = 0.002) (Table 6). Thus, age, BMI, and potato intake frequency were positively associated with TG levels in both men and women. In contrast, the frequency of alcohol intake was associated with TG levels only in men, whereas meat, oils, and fats were positively and negatively associated with TG levels only in women.

HDL-C in males was positively associated with age (0.09 (0.02, 0.17), *p* = 0.02) and the frequency of alcohol intake (0.067 (0.034, 0.10), *p* < 0.001) and negatively associated with BMI (−1.3 (−1.5, −1.1), *p* < 0.001) and the frequency of potato intake (−0.9 (−1.4, −0.3), *p* = 0.004) (Table 6). In females, HDL-C was positively associated with age (0.12 (0.06, 0.17), *p* < 0.001) and negatively associated with BMI (−1.2 (−1.3, −1.0), *p* < 0.001) (Table 6). Thus, sex, age, and frequency of alcohol intake were positively associated with HDL-C levels, whereas BMI was positively associated with HDL-C levels.

The non-HDL-C level in males was positively associated with age, BMI, frequency of dairy product consumption, and frequency of soft drink consumption. In females, non-HDL-C was positively associated with age (0.8 (0.6, 1.0), *p* < 0.001), BMI (2.8 (2.3, 3.4), *p* < 0.001), sleep duration (2.1 (0.2, 4.0), *p* = 0.04), frequency of dairy product intake (0.9 (0.1, 1.7), *p* = 0.03), and frequency of alcohol intake (0.06 (0.03, 0.09); *p* < 0.001) (Table 6). Non-HDL-C levels in females were positively associated with age, BMI, frequency of dairy product intake, and frequency of soft drink intake. In females, non-HDL-C was positively associated with age (1.0 (0.6, 1.0), *p* < 0.001), BMI (2.1 (1.7, 3.4), *p* < 0.001), and frequency of egg intake (0.6 (0.1, 1.0), *p* = 0.02) and negatively associated with frequency of meat intake (−0.4 (−0.6, −0.1), *p* = 0.02), frequency of soybean intake (−0.3 (−0.6, −0.03), *p* = 0.03), and frequency of alcohol intake (−1.3 (−1.9, −0.7), *p* < 0.001) (Table 6). Thus, age and BMI were positively associated with non-HDL-C levels in both men and women. In contrast, sleep duration and the frequency of dairy product intake were associated with non-HDL-C levels only in men. In women, the frequency of egg intake was positively associated with non-HDL-C levels, whereas the frequency of meat, soybean, and alcohol intake was negatively associated with non-HDL-C levels.

## 4. Discussion

In this study, we first examined whether the frequency of intake of 10-item foods, snacks, sugar-sweetened coffee/tea, soft drinks, and alcoholic beverages varied by age and sex in Japan. We then performed multivariate analysis in groups divided by sex to identify associations between food frequency and metabolic parameters, with special reference to age and sex differences. Consistent with the sex differences, the correlation between food frequency and metabolic phenotypes differed between males and females. Thus, since the effects of food frequency on body size and blood parameters are dependent on age and sex, dietary guidance may need to be based on sex differences in food preference.

First, we confirmed sex differences in food preferences between males and females. As expected, males preferred to eat meat, and females preferred to eat soybeans, dairy products, vegetables, and fruits. Males also preferred to drink soft drinks and alcohol, whereas females preferred to eat snacks. These results are consistent with those of previous studies [1,2]. It is interesting to observe the same differences in food preferences between men and women, regardless of race or culture. Food preferences may be based on taste rather than the type of food itself [2]. Thus, food preference is an important factor for understanding sex differences in eating habits. However, many guidelines for diabetes and dyslipidemia do not consider sex differences in food preference. Considering sex-related differences in food preferences will provide better dietary guidance to patients.

In this study, vegetables and meat were consumed daily, whereas fruits, seaweed, and potatoes were consumed less frequently. The consumption of fruits and soybeans by men and the consumption of meat and fish by women seem to be encouraged. Potatoes and seaweed are considered necessary sources of dietary fiber for both sexes. These findings suggest that awareness of the importance of green vegetable and meat consumption is widespread, but increasing awareness of the importance of the consumption of soy, fish, seaweed, fruits, and potatoes, which are rich in trace elements and vitamins, seems necessary. Moreover, dietary fiber intake and the consumption of fruits, soybeans, potatoes, and seaweed are low in Japan [26].

Aging also affects food preferences [27]. The frequency of meat and egg intake was independent of age in both males and females. In contrast, the frequency of fish, soybean, and dairy product intake increased with age in both males and females. These findings suggest that the frequency of protein intake increases with age, independent of sex. The frequency of seaweed, fruit, vegetable, and potato intake also increased with age. These findings suggest that the frequency of dietary fiber intake increases with age, independent of sex. It is likely that many individuals consider the role of food in their health during aging. Taken together, these findings indicate that dietary variety increases with age. Many healthy diets, predominantly containing nutrient-rich plant foods and limited as to red and processed meats, have been associated with lower mortality and longevity [28]. However, the frequency of seaweed, potato, and fruit intake was lower than the frequency of meat and vegetable intake. We need to educate people about the benefits of consuming seaweed, fruits, and potatoes as sources of dietary fiber.

Sleep duration was also negatively correlated with HbA1c levels independent of sex. A long sleep duration (≥9 h/day) is associated with higher HbA1c levels in patients with type-2 diabetes (T2D) on glucose-lowering medications [29]. Waist circumference was also negatively associated with sleep duration, which was inconsistent with our data [30]. Thus, to increase sleep duration, it is necessary to improve the working environment and educate people on the importance of not staying late in their daily lives.

The relationships between the frequency of food intake and metabolic parameters differed between males and females. In males, meat intake was positively associated with BMI and HbA1c and negatively associated with eGFR. In contrast, the frequency of meat intake in females was correlated with waist circumference and TG levels. These findings suggest that individuals who frequently consume meat should receive dietary guidance to prevent diabetes (males) and dyslipidemia (females).

Similarly, dairy products had opposite effects on males and females. In males, dairy product intake was positively correlated with BMI, eGFR, and non-HDL-C levels. In females, dairy product intake was negatively associated with eGFR and UA levels. Consistently, some studies have reported that the risk of hyperuricemia and gout is negatively associated with the intake of dairy products or soy foods [31]. In terms of renal function, dairy products had the opposite association with eGFR in both men and women. Although no distinction was made between low-fat dairy products and regular dairy products in this study, low-fat dairy products have been reported to prevent renal dysfunction [32]. It is possible that there is a difference in the frequency of the consumption of low-fat and regular dairy products between men and women.

Fish, egg, soybean, and fruit intake were correlated with metabolic parameters only in females. Fish intake was positively correlated with BMI and eGFR. In the middle-aged to older Chinese population, higher fish and seafood consumption (≥11 portions/week) was found to be associated with a greater eGFR in participants with diabetes, whereas lower fish consumption was associated with a greater eGFR in participants without diabetes [33]. However, the differences between groups, in terms of the frequency of fish consumption 0–3, 4–6, and 7–10, were not significant [33]. The participants in this study were more likely to consume fish and seafood thrice a week, and most of them did not have diabetes. Thus, the differences in the results may be due to differences in eating habits and sex.

Egg intake was positively associated with BMI, waist circumference, and non-HDL-C level only in females. Some studies have reported that, among U.S. adults, increased consumption of dietary cholesterol or eggs is significantly associated with an increased risk of incident cardiovascular disease and all-cause mortality in a dose-dependent manner [34]. In contrast, in healthy Japanese individuals, there was no association between the daily consumption of one egg and blood cholesterol levels [35]. Blood non-HDL-C levels increased to levels comparable to those in overweight and underweight women aged 50–65 years [36]. Therefore, care should be taken to avoid excessive egg consumption by women of all ages.

Soybean intake was negatively associated with BMI, waist circumference, and non-HDL-C level only in females. Soy may be a suitable food for antiobesity efforts because of its high protein and isoflavone contents [37,38,39]. Women who consumed more soy during adulthood had a lower BMI, but this relationship was primarily observed in Caucasian and postmenopausal women [37]. Moreover, in hypercholesterolemic postmenopausal women fed a National Cholesterol Education Program Step I diet with isolated soy protein and moderate amounts of isoflavones, non-HDL-C levels were reduced compared with those in the control group (*p* < 0.05) [38]. In Japan, a higher intake of fermented soy is associated with a lower risk of mortality [40,41]. A higher intake of total phytoestrogens, including isoflavones, lignans, and coumarins, and foods rich in these compounds was associated with a lower risk of total and cause-specific mortality in generally healthy adults in the U.S. [42]. Thus, these findings suggest that individuals who frequently consume soybeans should receive dietary guidance to prevent obesity and dyslipidemia (especially in females).

Fruit intake correlated with BMI only in females. This trend was also observed in males. Similarly, a relationship between fruit intake and BMI has also been reported [43,44]. Some studies have reported significant associations between higher fruit consumption and lower BMI among female university students [43]. Greater fruit intake is associated with a lower risk of being overweight or obese in middle-aged and older women [44]. Therefore, fruit intake may effectively prevent weight gain, particularly among women.

Sugar intake has been associated with obesity and dental caries. Sugar intake is recommended to constitute no more than 10% of total energy intake, according to the World Health Organization guidelines [45]. However, as the amount of sugar used in the diet cannot be quantified, there is no mention of quantity limits in the Japanese Dietary Intake Standards [26]. The amount of free sugar was estimated on the basis of the frequency of the consumption of snacks, sugar-sweetened coffee, tea, and other soft drinks. According to our data, the frequency of snack consumption was greater in females than in males, and the frequency of soft drink consumption was greater in males. When assessing sugar intake, particular attention should be given to the frequency of soft drink and snack consumption by men and women, respectively.

In this study, the average alcohol consumption among the participants was 1.3 times per week. However, the frequency per week significantly increased with age in both men and women. After adjusting for age and BMI, alcohol intake correlated with UA and HDL-C levels in both males and females. Many studies have reported that hyperuricemia, characterized by high UA levels, may occur due to increased UA production or reduced UA excretion [46]. A positive association between alcohol intake and HDL-C levels has also been reported by many researchers [47], but an increase in HDL-C levels is not necessarily good [48,49]. Some studies have reported that high-density lipoprotein function in binge drinkers is impaired, with lower paraoxonase activity and greater glycation [48]. In males, TG levels were associated with alcohol intake, which is consistent with previous findings [49]. Thus, drinking alcoholic beverages is harmful to UA, HDL-C, and TG levels in males.

In contrast, in females, alcohol intake was positively associated with handgrip strength and negatively associated with the HbA1c level. Several studies have shown that handgrip strength significantly increases with increased daily alcohol consumption in both sexes [50]. Our data also revealed a correlation between the frequency of alcohol consumption and handgrip strength, with a significant difference in women (*p* = 0.007) and a trend toward significance in men (*p* = 0.07). The underlying mechanism of the relationship between the frequency of alcohol consumption and grip strength is unknown, but the results are reproducible [51]. Alcohol intake was negatively associated with HbA1c levels only in females. Consistently, higher alcohol intake was associated with lower HbA1c levels, even after adjusting for confounding factors such as age, sex, and fasting glucose concentration in a nationally representative sample of Korean adults [52]. Importantly, most Japanese people drink infrequently (once per week) [53]. Thus, the frequency of alcohol consumption had a positive effect on grip strength and HbA1c levels. Therefore, although our population consumes alcohol relatively infrequently compared with the overall Japanese population, the advantages and disadvantages of drinking alcoholic beverages should be fully considered.

This study has several limitations. This was an observational study. Therefore, causality could not be demonstrated. Although it was not possible to determine a causal relationship, this study advances the previously reported relationship between diet and metabolic parameters according to sex. In addition, prospective studies are needed to determine the effectiveness of nutritional guidance in terms of sex-based differences. We did not check for information regarding the participants’ menstrual cycles or lifestyle factors (exercise and smoking). Some nurses and nursing assistants walk 20,000 steps per day for their jobs, and pedometers should be used to investigate the number of steps. The sleep duration was self-reported. Another limitation of this study was the lack of information on medication use and medical history.

## 5. Conclusions

Sex-related differences were observed in the frequency of food intake and its effect on metabolic parameters. These findings indicate that the food restrictions in the guidelines should not be uniformly applied regardless of sex. For example, non-HDL-C was significantly positively correlated with the frequency of egg intake only in women, even though egg intake frequency was the same in men and women. Although there is no evidence that women like omelets and scrambled eggs more than men, we need to look at even the way the eggs are prepared since butter is added to scrambled eggs, and nothing is added to boiled eggs.

In contrast, the effects of alcohol intake on UA levels were similar in men and women. The frequency of potato consumption was also significantly correlated with triglycerides in both men and women. Regardless of sex, alcohol and potatoes (starch) are converted directly to uric acid and triglycerides in the body. Dietary restriction of these related foods is considered to be effective for both sexes.

We decided to conduct this study because we believe that nutrition education in the workplace can improve employee health. On the basis of these study data, our university will promote nutrition education to employees by creating cooking recipes that supplement deficient food groups (fish, soybeans, fruits, etc.) and offering cooking classes in the workplace.

Moreover, we need to test whether dietary restrictions influenced by sex have a beneficial effect on the improvement of blood markers (HbA1c, lipids, and eGFR). Prospective studies are necessary to implement nutritional guidance that considers sex-related differences.

## Figures and Tables

**Table 1:** Background of the subjects.

	Total (n = 3147)	Male (n = 968)	Female (n = 2179)	*p*
Age	35.1 (11.3)	37.9 (10.9)	33.9 (11.2)	**<0.001** *
BMI	21.9 (3.4)	23.3 (3.4)	21.3 (3.3)	**<0.001**
Waist circumference	74.0 (10.3)	81.2 (9.1)	70.8 (9.1)	**<0.001**
Handgrip strength	29.6 (10.9)	41.1 (9.1)	24.5 (7.1)	**<0.001**
HbA1c	5.42 (0.38)	5.46 (0.42)	5.40 (0.35)	**<0.001**
Triglyceride	101.3 (73.3)	131.5 (95.6)	87.8 (55.8)	**<0.001** *
HDL-C	62.1 (14.4)	53.7 (12.4)	65.8 (13.6)	**<0.001**
Non-HDL-C	128.9 (31.9)	139.2 (33.8)	124.4 (29.9)	**<0.001**
eGFR	90.3 (17.6)	84.7 (15.8)	92.8 (17.7)	**<0.001**
Uric acid	4.8 (1.3)	6.0 (1.2)	4.2 (0.9)	**<0.001**
Hours of sleep	6.4 (1.0)	6.3 (1.0)	6.4 (1.0)	**0.001**
Meat (/week)	8.2 (4.2)	8.8 (4.5)	7.8 (4.1)	**<0.001** *
Fish (/week)	3.5 (2.7)	3.8 (3.0)	3.3 (2.6)	**0.001** *
Egg (/week)	4.2 (2.7)	4.2 (3.0)	4.2 (2.5)	0.58 *
Soybeans(/week)	5.8 (4.3)	5.5 (4.3)	5.9 (4.3)	**0.002** *
Dairy products (/week)	3.2 (2.8)	2.9 (2.8)	3.3 (2.8)	**<0.001** *
Seaweed (/week)	2.0 (2.1)	1.9 (2.2)	2.0 (2.0)	0.72 *
Vegetables (/week)	9.2 (4.7)	8.9 (4.8)	9.3 (4.7)	**0.029** *
Fruits (/week)	2.9 (2.6)	2.7 (2.7)	3.1 (2.5)	**<0.001** *
Potatoes (/week)	2.1 (1.7)	2.0 (1.8)	2.1 (1.7)	0.24 *
Oils and fats (/week)	10.0 (5.5)	10.1 (5.5)	9.9 (5.6)	0.11 *
Snacks (/week)	11.0 (12.0)	9.4 (11.9)	11.7 (12.0)	**<0.001** *
Coffee/tea with sugar (/week)	0.9 (2.7)	0.9 (2.8)	0.9 (2.7)	0.057 *
Soft drinks (/week)	1.3 (2.6)	1.9 (3.0)	1.0 (2.3)	**<0.001** *
Alcohol (/week)	1.3 (2.1)	1.7 (2.4)	1.1 (1.9)	**<0.001** *

The data are presented as the mean (SD). * Mann–Whitney U test. Bold letters indicate significance (*p* < 0.05 vs. male). Abbreviations: BMI, body mass index; HbA1c, glycated hemoglobin A1c; HDL-C, high-density lipoprotein cholesterol; eGFR, estimated glomerular filtration rate.

**Table 2:** Frequency of the intake of ten food items by sex and age group.

		Male			Female	
Foods	Age	Mean (SD)	*p*	Age	Mean (SD)	*p*
Meat	20 yo (n = 303)	8.7(4.0)	0.76	20 yo (n = 1091)	7.7(0.1)	**0.02**
	30 yo (n = 253)	8.6(4.4)		30 yo (n = 399)	8.2(0.2)	
	40 yo (n = 222)	9.1(4.8)		40 yo (n = 383)	8.3(0.2)	
	50 yo (n = 190)	8.9(4.8)		50 yo (n = 306)	7.5(0.2)	
Fish	20 yo (n = 303)	3.3(3.0)	**<0.001**	20 yo (n = 1091)	3.0(0.1)	**<0.001**
	30 yo (n = 253)	3.7(2.9)		30 yo (n = 399)	3.4(0.1) ***	
	40 yo (n = 221)	3.7(2.9) *		40 yo (n = 383)	3.6(0.1) ***	
	50 yo (n = 189)	4.7(3.3) ***		50 yo (n = 306)	4.0(0.1) ***	
Egg	20 yo (n = 303)	4.3(2.8)	0.4	20 yo (n = 1091)	4.1(0.1)	0.9
	30 yo (n = 253)	4.1(2.8)		30 yo (n = 399)	4.2(0.1)	
	40 yo (n = 221)	4.3(3.7)		40 yo (n = 383)	4.3(0.1)	
	50 yo (n = 189)	4.2(2.9)		50 yo (n = 306)	4.2(0.1)	
Soybean	20 yo (n = 303)	4.8(4.2)	**<0.001**	20 yo (n = 1091)	5.2(0.1)	**<0.001**
	30 yo (n = 253)	5.4(4.3)		30 yo (n = 399)	6.0(0.2) **	
	40 yo (n = 221)	6.0(4.4) **		40 yo (n = 383)	6.8(0.2) ***	
	50 yo (n = 189)	5.9(4.1) **		50 yo (n = 306)	7.3(0.3) ***	
Dairy product	20 yo (n = 303)	2.5(2.4)	**<0.001**	20 yo (n = 1091)	2.9(0.1)	**<0.001**
	30 yo (n = 253)	2.7(3.1)		30 yo (n = 399)	3.4(0.1) **	
	40 yo (n = 221)	3.2(2.9) *		40 yo (n = 383)	3.5(0.1) ***	
	50 yo (n = 189)	3.4(2.8) **		50 yo (n = 306)	4.0(0.2) ***	
Seaweed	20 yo (n = 303)	1.4(1.5)	**<0.001**	20 yo (n = 1091)	1.6(0.06)	**<0.001**
	30 yo (n = 253)	1.9(2.5) *		30 yo (n = 399)	1.9(0.09) **	
	40 yo (n = 221)	2.2(2.5) ***		40 yo (n = 383)	2.5(0.1) ***	
	50 yo (n = 189)	2.6(2.0) ***		50 yo (n = 306)	2.6(0.1) ***	
Vegetable	20 yo (n = 303)	8.0(4.4)	**<0.001**	20 yo (n = 1091)	8.8(0.1)	**<0.001**
	30 yo (n = 253)	8.6(4.6) *		30 yo (n = 399)	9.3(0.2)	
	40 yo (n = 221)	9.4(4.8) ***		40 yo (n = 383)	9.4(0.2)	
	50 yo (n = 189)	10.1(5.2) ***		50 yo (n = 306)	10.7(0.3) ***	
Fruit	20 yo (n = 303)	1.9(2.2)	**<0.001**	20 yo (n = 1091)	2.6(0.07)	**<0.001**
	30 yo(n = 253)	2.6(3.0) **		30 yo (n = 399)	3.4(0.1) ***	
	40 yo(n = 221)	2.7(2.4) ***		40 yo (n = 383)	3.4(0.1) ***	
	50 yo(n = 189)	3.9(2.9) ***		50 yo (n = 306)	3.9(0.1) ***	
Potatoes	20 yo (n = 303)	1.8(1.6)	**0.022**	20 yo (n = 1091)	1.9(0.1)	**<0.001**
	30 yo (n = 253)	2.1(2.4)		30 yo (n = 399)	2.3(0.1) **	
	40 yo (n = 221)	2.2(1.7)*		40 yo (n = 383)	2.3(0.1) ***	
	50 yo (n = 189)	2.1(1.6) *		50 yo (n = 306)	2.2(0.1) ***	
Oils and fats	20 yo (n = 303)	8.8(4.7)	**<0.001**	20 yo (n = 1091)	8.3(0.1)	**<0.001**
	30 yo (n = 253)	9.7(5.4)		30 yo (n = 399)	10.6(0.3) ***	
	40 yo (n = 221)	11.1(5.5) ***		40 yo (n = 383)	11.5(0.3) ***	
	50 yo (n = 189)	11.7(6.1) ***		50 yo (n = 306)	12.8(0.3) ***	

The data are presented as the mean (SD). Bold letters indicate significance (* *p* < 0.05 vs. 20 yo, ** *p* < 0.01 vs. 20 yo, *** *p* < 0.001 vs. 20 yo).

**Table 3:** Food preferences by age and sex groups.

		Male			Female	
Foods	Age	Mean (SE)	*p*	Age	Mean (SE)	*p*
Snacks	20 yo (n = 303)	8.0 (10.0)	**<0.001**	20 yo (n = 1091)	10.5 (10.3)	**<0.001**
	30 yo (n = 253)	9.1 (16.1)		30 yo (n = 399)	10.9 (0.8)	
	40 yo (n = 221)	10.9 (10.5) **		40 yo (n = 383)	13.5 (14.7) **	
	50 yo (n = 189)	10.2 (9.0) **		50 yo (n = 306)	14.8 (14.6) **	
Coffee/Tea with sugars	20 yo (n = 303)	0.5 (1.4)	0.42	20 yo (n = 1091)	0.7 (1.9)	0.15
	30 yo (n = 253)	1.0 (3.5)		30 yo (n = 399)	1.3 (3.8)	
	40 yo (n = 221)	1.3 (3.2)		40 yo (n = 383)	1.0 (2.7)	
	50 yo (n = 189)	0.9 (2.8)		50 yo (n = 306)	0.9 (3.0)	
Soft Drink	20 yo (n = 303)	2.0 (3.1)	0.32	20 yo (n = 1091)	1.0 (2.3)	0.09
	30 yo (n = 253)	2.0 (3.5)		30 yo (n = 399)	1.1 (2.0)	
	40 yo (n = 221)	1.7 (2.5)		40 yo (n = 383)	1.0 (2.9)	
	50 yo (n = 189)	1.7 (2.5)		50 yo (n = 306)	0.8 (1.7)	
Alcohol	20 yo (n = 303)	1.3 (2.4)	**<0.001**	20 yo (n = 1091)	0.8 (1.2)	**<0.001**
	30 yo (n = 253)	1.5 (2.0)		30 yo (n = 399)	1.1 (2.0)	
	40 yo (n = 221)	2.3 (2.6) ***		40 yo (n = 383)	1.6 (2.3) **	
	50 yo (n = 189)	2.2 (2.5) ***		50 yo (n = 306)	1.9 2.6) ***	

The data are presented as the mean (SD). Bold letters indicate significance ( ** *p* < 0.01 vs. 20 yo, *** *p* < 0.001 vs. 20 yo).

**Table 4:** The effects of food frequency on circumference and handgrip strength.

	Waist Circumference				Handgrip Strength			
	Male		Female		Male		Female	
	B	*p*	B	*p*	B	*p*	B	*p*
Meat	0.05 (−0.04,0.13)	0.27	**0.09** **(0.02, 0.16)**	**0.01**	−0.04 (−0.18, 0.10)	0.56	−0.01 (−0.09, 0.07)	0.74
Fish	−0.09 (−0.21, 0.04)	0.16	−0.11(−0.23,0.003)	0.06	-0.05 (−0.26, 0.17)	0.67	-0.07 (−0.2, 0.06)	0.31
Egg	−0.004 (−0.12, 0.11)	0.95	**0.19** **(0.07, 0.30)**	**0.001**	0.07 (−0.13, 0.27)	0.5	0.12 (−0.004, 0.25)	0.058
Soybeans	0.02 (-0.07, 0.10)	0.74	**−0.17** **(−0.17, −0.03)**	**0.007**	0.08 (−0.07, 0.23)	0.3	−0.001 (−0.08, 0.08)	0.98
Dairy products	**−0.17** **(−0.3, −0.04)**	**0.01**	0.05 (−0.06, 0.15)	0.37	0.06 (−0.07, 0.29)	0.61	0.11 (−0.004, 0.22)	0.058
Seaweed	−0.03 (−0.22, 0.15)	0.72	0.14 (−0.003,0.39)	0.055	0.07 (−0.25, 0.38)	0.69	**0.23** **(0.07, 0.4)**	**0.005**
Vegetables	−0.02 (−0.1, 0.1)	0.67	−0.02 (−0.09, 0.04)	0.49	0.004 (−0.14, 0.15)	0.96	−0.05 (−0.13, 0.02)	0.17
Fruits	0.1 (−0.1, 0.2)	0.52	−0.03 (−0.15, 0.09)	0.67	0.21 (−0.5, 0.46)	0.11	−0.085 (−0.22, 0.05)	0.21
Potatoes	0.02 (−0.2, 0.3)	0.85	0.13 (−0.05, 0.3)	0.15	−0.39 (−0.79, 0.01)	0.054	−0.1 (−0.3, 0.1)	0.32
Oils and fats	**0.08** **(0.01, 0.15)**	**0.02**	0.05 (−0.002,0.11)	0.057	0.05 (−0.07, 0.17)	0.41	0.03 (−0.03, 0.09)	0.37
Snacks	0.002 (−0.03, 0.04)	0.92	−0.01 (−0.03, 0.02)	0.51	−0.03 (−0.09, 0.03)	0.33	0.011 (−0.01, 0.04)	0.39
Coffee/tea with sugar	0.05 (−0.08, 0.18)	0.42	0.04 (−0.07, 0.14)	0.49	−0.13 (−0.35, 0.09)	0.23	0.01 (−0.11, 0.12)	0.89
Soft drinks	0.03 (−0.1, 0.15)	0.68	0.12 (−0.001, 0.23)	0.052	−0.05 (−0.26, 0.15)	0.61	0.05 (−0.09, 0.18)	0.5
Alcohol	-0.05 (−0.19, 0.09)	0.48	−0.05 (−0.19, 0.10)	0.54	0.22 (−0.02, 0.46)	0.07	**0.23** **(0.06, 0.39)**	**0.007**
Age	**0.09** **(0.05, 0.12)**	**<0.001**	**0.24** **(0.21, 0.26)**	**<0.001**	**−0.1** **(−0.15, −0.04)**	**<0.001**	0.01 (−0.02, 0.04)	0.48
BMI	**2.1** **(2.0, 2.2)**	**<0.001**	**1.66** **(1.58, 1.75)**	**<0.001**	**0.7** **(0.5, 0.8)**	**<0.001**	**0.14** **(0.05, 0.23)**	**0.003**
Hours of sleep	0.3 (−0.1, 0.6)	0.13	**0.29** **(0.03, 0.54)**	**0.03**	−0.2 (−0.8, 0.4)	0.5	−0.2 (−0.5, 0.1)	0.27

The gray areas (letters in bold) indicate significance (*p* < 0.05).

**Table 5:** The effects of food frequency on HbA1c, eGFR, and uric acid.

	HbA1c				eGFR			
	Male		Female		Male		Female	
	B	*p*	B	*p*	B	*p*	B	*p*
Meat	**0.007** **(0.001, 0.013)**	**0.03**	0.003 (0, 0.007)	0.09	**−0.3** **(−0.5, −0.1)**	**0.01**	−0.09 (−0.25, 0.08)	0.32
Fish	0.004 (−0.005, 0.013)	0.35	−0.001 (−0.006, 0.005)	0.83	−0.01 (−0.3, 0.3)	0.97	**0.4** **(0.1, 0.7)**	**0.005**
Egg	−0.007 (−0.016, 0.001)	0.097	0 (−0.005, 0.006)	0.87	0.2 (−0.1, 0.5)	0.15	0.1 (−0.1, 0.4)	0.31
Soybeans	−0.006 (−0.012, 0.001)	0.08	0.001 (−0.003, 0.004)	0.76	0.1 (−0.1, 0.3)	0.29	−0.08 (−0.25, 0.09)	0.37
Dairy products	0.007 (−0.003, 0.016)	0.18	0.003 (−0.002, 0.008)	0.21	0.2 (−0.1, 0.6)	0.15	**−0.3** **(−0.5, −0.02)**	**0.03**
Seaweed	−0.005 (−0.019, 0.009)	0.49	−0.005 (−0.012, 0.002)	0.19	**0.8** **(0.3, 1.2)**	**0.001**	−0.07 (−0.4, 0.3)	0.69
Vegetables	0.004 (−0.002, 0.01)	0.19	−0.001 (−0.005, 0.002)	0.45	−0.1 (−0.3, 0.2)	0.63	−0.01 (−0.17, 0.15)	0.9
Fruits	−0.004 (−0.015, 0.007)	0.47	0.004 (−0.002, 0.010)	0.22	−0.04 (−0.4, 0.3)	0.82	−0.05 (−0.3, 0.2)	0.73
Potatoes	0.014 (−0.003, 0.031)	0.11	0.005 (−0.004, 0.014)	0.26	**−0.9** **(−1.4, −0.3)**	**0.004**	0.1 (−0.3, 0.5)	0.64
Oils and fats	−0.002 (−0.008, 0.003)	0.34	0 (−0.003, 0.014)	0.85	−0.02 (−0.2, 0.2)	0.84	−0.02 (−0.15, 0.12)	0.82
Snacks	−0.001 (−0.003, 0.002)	0.48	0.001 (−0.001, 0.002)	0.3	−0.06 (−0.15, 0.02)	0.15	0.002 (−0.05, 0.06)	0.94
Coffee/tea with sugar	−0.001 (−0.011, 0.008)	0.82	−0.002 (−0.007, 0.003)	0.47	−0.1 (−0.5, 0.2)	0.37	−0.16 (−0.4, 0.08)	0.2
Soft drinks	0.005 (−0.004, 0.014)	0.27	0.002 (−0.004, 0.008)	0.51	0.3 (−0.1, 0.5)	0.14	0.1 (−0.2, 0.4)	0.58
Alcohol	−0.01 (−0.02, 0.002)	0.11	**−0.018** **(−0.025, −0.01)**	**<0.001**	0.2 (−0.2, 0.5)	0.29	−0.04 (−0.39, 0.32)	0.84
Age	**0.011** **(0.009, 0.014)**	**<0.001**	**0.01** **(0.009, 0.012)**	**<0.001**	**−0.8** **(−0.9, −0.7)**	**<0.001**	**−0.83** **(−0.89, −0.76)**	**<0.001**
BMI	**0.031** **(0.024, 0.038)**	**<0.001**	**0.02** **(0.016, 0.024)**	**<0.001**	−0.1 (−0.4, 0.1)	0.3	−0.1 (−0.3, 0.1)	0.33
Hours of sleep	**−0.04** **(−0.064, −0.016)**	**0.001**	**−0.03** **(−0.04, −0.01)**	**<0.001**	−0.4 (−1.2, 0.4)	0.35	0.3 (−0.3, 0.9)	0.32
	**Uric Acid**							
	**Male**				**Female**			
	**B**		***p***		**B**		***p***	
Meat	0.013 (−0.005, 0.03)		0.14		0.003 (−0.006, 0.013)		0.48	
Fish	−0.01 (−0.04, 0.02)		0.49		−0.002 (−0.018, 0.014)		0.82	
Egg	−0.004 (−0.03, 0.02)		0.77		−0.012 (−0.027, 0.003)		0.12	
Soybeans	0.01 (−0.01, 0.03)		0.4		**0.013** **(0.003, 0.023)**		**0.009**	
Dairy products	−0.01 (−0.04, 0.02)		0.71		**−0.01** **(−0.03, 0)**		**0.048**	
Seaweed	−0.04 (−0.08, 0.01)		0.09		0.01 (−0.01, 0.03)		0.41	
Vegetables	0.01 (−0.01, 0.03)		0.43		0.005 (−0.004, 0.015)		0.25	
Fruits	−0.03 (−0.06, 0.01)		0.14		−0.012 (−0.028, 0.004)		0.15	
Potatoes	0.03 (−0.02, 0.08)		0.29		0.023 (−0.001, 0.047)		0.07	
Oils and fats	−0.002 (−0.02, 0.01)		0.8		**−0.01** **(−0.02, −0.002)**		**0.01**	
Snacks	0 (−0.008, 0.007)		0.92		−0.002 (−0.005, 0.001)		0.17	
Coffee/tea with sugar	0.02 (−0.01, 0.05)		0.26		−0.002 (−0.016, 0.012)		0.79	
Soft drinks	0.01 (−0.02, 0.04)		0.37		0.005 (−0.011, 0.021)		0.51	
Alcohol	**0.06** **(0.03, 0.09)**		**<0.001**		**0.06** **(0.04, 0.08)**		**<0.001**	
Age	−0.002 (−0.009, 0.005)		0.6		0.002 (−0.002, 0.006)		0.31	
BMI	**0.11** **(0.09, 0.13)**		**<0.001**		**0.084** **(0.073, 0.096)**		**<0.001**	
Hours of sleep	−0.02 (−0.09, 0.05)		0.58		−0.01 (−0.05,0.02)		0.42	

The gray areas (letters in bold) indicate significance (*p* < 0.05). Abbreviations: HbA1c, glycated hemoglobin A1c; eGFR, estimated glomerular filtration.

**Table 6:** Effects of food frequency on plasma lipid levels.

TG					HDLc				non-HDLc			
	Male		Female		Male		Female		Male		Female	
	B	*p*	B	*p*	B	*p*	B	*p*	B	*p*	B	*p*
Meat	0.3 (−1.1, 1.7)	0.7	**0.6** **(0.04, 1.2)**	**0.04**	0.2 (−0.01, 0.4)	0.06	−0.1 (−0.2, 0.1)	0.35	−0.1 (−0.6, 0.4)	0.65	**−0.4** **(−0.6, −0.1)**	**0.02**
Fish	−1.3 (−3.5, 0.9)	0.25	0.4 (−0.5,1.4)	0.38	−0.1 (−0.4, 0.1)	0.33	0.1 (−0.2, 0.3)	0.63	−0.2 (−1.0, 0.6)	0.6	0.1 (−0.4, 0.5)	0.84
Egg	−0.3 (−2.3, 1.8)	0.8	0.8 (−0.1,1.8)	0.07	0.2 (−0.1,0.5)	0.12	0.3 (0.1, 0.5)	0.02	−0.2 (−0.9, 0.5)	0.62	**0.6** **(0.1, 1.0)**	**0.02**
Soybeans	−0.7 (−2.3, 0.8)	0.36	−0.4 (−1.0, 0.2)	0.2	0.2 (−0.04, 0.4)	0.12	0.12 (−0.03, 0.26)	0.12	−0.4 (−1.0, 0.1)	0.11	**−0.3** **(−0.6, −0.03)**	**0.03**
Dairy products	2.2 (−0.1, 4.5)	0.07	−0.5 (−1.3, 0.3)	0.24	0.004 (−0.3, 0.3)	0.98	−0.01 (−0.21, 0.2)	0.94	**0.9** **(0.1, 1.7)**	**0.03**	0.06 (−0.4, 0.5)	0.78
Seaweed	0.2 (−3.0, 3.4)	0.9	0.3 (−0.9, 1.5)	0.67	-0.1 (−0.5,0.3)	0.65	−0.2 (−0.5, 0.1)	0.16	0.2 (−0.9, 1.3)	0.76	−0.5 (−1.1, 0.1)	0.13
Vegetables	0.3 (−1.2, 1.7)	0.73	0.1 (−0.5,0.6)	0.8	-0.2 (−0.4, 0)	0.09	0.1 (−0.04, 0.2)	0.16	0.3 (−0.3, 0.8)	0.34	0.1 (−0.2, 0.4)	0.58
Fruits	−2.2 (−4.8, 0.4)	0.1	0.6 (−0.4, 1.5)	0.27	−0.1 (−0.4, 0.3)	0.7	−0.2 (−0.5, 0.03)	0.09	−0.4 (−1.3, 0.5)	0.36	0.1 (−0.4, 0.7)	0.57
Potatoes	**5.0** **(0.9, 9.0)**	**0.02**	**1.8** **(0.3, 3.2)**	**0.02**	−0.1 (−0.6, 0.4)	0.71	−0.4 (−0.8, −0.04)	0.03	0.3 (−1.1, 1.7)	0.7	0.3 (−0.4, 1.0)	0.42
Oils and fats	−0.8 (−2, 0.4)	0.2	**−0.7** **(−1.2, −0.3)**	**0.002**	−0.02 (−0.2, 0.1)	0.8	0.1 (−0.1, 0.2)	0.25	−0.3 (−0.7, 0.1)	0.11	0.01 (−0.2, 0.2)	0.95
Snacks	−0.2 (−0.8, 0.5)	0.63	−0.02 (−0.2, 0.2)	0.87	0.01 (−0.1, 0.1)	0.78	0.06 (0.01, 0.1)	0.02	0.1 (−0.1, 0.3)	0.27	0.06 (−0.03, 0.16)	0.21
Coffee/tea with sugar	2.2 (−0.04, 4.4)	0.054	0.3 (−0.5, 1.2)	0.43	−0.1 (−0.4, 0.1)	0.32	−0.2 (−0.4, 0.1)	0.17	0.4 (−0.4, 1.2)	0.32	−0.1 (−0.5, 0.4)	0.72
Soft drinks	1.3 (−0.8, 3.4)	0.23	0.4 (−0.6, 1.4)	0.41	−0.2 (−0.4, 0.1)	0.24	−0.04 (−0.13, 0.2)	0.78	−0.5 (−1.2, 0.3)	0.21	−0.4 (−0.9, 0.1)	0.09
Alcohol	**3** **(0.5, 5.4)**	**0.02**	−0.9 (−2.1, 0.3)	0.15	**1.0** **(0.7, 1.3)**	**<0.001**	**1.3** **(1.0, 1.6)**	**<0.001**	−0.7 (−1.5, 0.2)	0.12	**−1.3** **(−1.9, −0.7)**	**<0.001**
Age	**1.1** **(0.5, 1.7)**	**<0.001**	**1.2** **(1.0, 1.4)**	**<0.001**	**0.09** **(0.02, 0.17)**	**0.014**	**0.12** **(0.06, 0.17)**	**<0.001**	**0.8** **(0.6, 1.0)**	**<0.001**	**1** **(0.9)**	**<0.001**
BMI	**7.6** **(5.9, 9.4)**	**<0.001**	**4.4** **(3.7, 5.0)**	**<0.001**	**−1.3** **(−1.5, −1.1)**	**<0.001**	**−1.2** **(−1.3, −1.0)**	**<0.001**	**2.8** **(2.3, 3.4)**	**<0.001**	**2.1** **(1.7, 2.4)**	**<0.001**
Hours of sleep	1.8 (−3.9, 7.5)	0.53	0.4 (−1.7, 2.5)	0.7	−0.1 (−0.8, 0.7)	0.88	−0.2 (-0.7, 0.3)	0.4	**2.1** **(0.2, 4.0)**	**0.04**	0.3 (−0.7, 1.4)	0.54

The gray areas (letters in bold) indicate significance (*p* < 0.05). Abbreviations: TG, triglyceride; HDLc, high-density cholesterol; non-HDLc, non-high-density cholesterol.

## Data Availability

The datasets used and/or analyzed during the current study will be made available by the corresponding author upon reasonable request.
